# Acute Toxicities of Bromophenols to Alga and Daphina: Comparative Species Sensitivity Distribution Between Standard and Dietary Supplementation Tests

**DOI:** 10.1155/jt/3399746

**Published:** 2025-05-11

**Authors:** Bin Li, Xueling Xiang, Jianghong Shi, Mengtao Zhang, Hui Ge

**Affiliations:** ^1^School of Environment, Harbin Institute of Technology, Harbin, China; ^2^School of Environmental Science and Engineering, Southern University of Science and Technology, Shenzhen, China

**Keywords:** acute toxicity, bromophenols, dietary supplementation, interspecies correlation estimation, species sensitivity distribution

## Abstract

Bromophenols are synthesized chemicals that are widely used in various industrial activities and are also naturally produced by marine algae as secondary metabolites, including 2,4-dibromophenol (2,4-DBP), 2,6-dibromophenol (2,6-DBP), and 2,4,6-tribromophenol (2,4,6-TBP). However, the toxicological profiles and toxicity data of these bromophenols remain largely unreported, necessitating further investigation. Acute toxicity tests of 2,4-DBP, 2,6-DBP, and 2,4,6-TBP were conducted in this study using *Scenedesmus quadricauda* and *Daphnia magna* (standard tests). Furthermore, a modified acute toxicity test of *D. magna* was proposed, which further evaluates the dietary supplementation effects (1.0 × 10^4^ cells/mL of *S. quadricauda*) on the toxicities of these three bromophenols (modified tests). The median effect concentrations (EC_50_s) of *D. magna* increased significantly when *S. quadricauda* was supplied as the dietary supplement. The EC_50_ values of 2,4-DBP increased from 2.17 to 4.47 mg/L, 2,6-DBP from 2.78 to 6.75 mg/L and 2,4,6-TBP from 1.57 to 3.28 mg/L. Moreover, the web-based interspecies correlation estimation platform coupled with the species sensitivity distribution model (Web-ICE-SSD) was used to calculate the fifth percentile hazard concentrations (HC_5_s) for 2,4-DBP, 2,6-DBP, and 2,4,6-TBP. The HC_5_ values when using standard test data for 2,4-DBP, 2,6-DBP, and 2,4,6-TBP were 0.55, 0.71, and 0.43 mg/L, respectively. In contrast, the HC_5_ values when using modified test data increased to 1.20, 1.80, and 0.88 mg/L. These results indicated that dietary supplementation during acute toxicity tests may provide more environment-related risk assessment.

## 1. Introduction

Bromophenols are extensively used in various industrial sectors. 2,4,6-tribromophenol (2,4,6-TBP) is used as a wood preservative due to its antifungal effects [1]. 2,4-dibromophenol (2,4-DBP) and 2,4,6-TBP are the primary intermediates for synthesizing tetrabromobisphenol A (TBBPA) and 1,2-bis(2,4,6-tribromophenoxy) ethane (BTBPE) [2]. Traditional brominated flame retardants (BFRs) such as polybrominated diphenyl ethers (PBDEs) are banned; hence, phenolic and epoxy resins use bromophenols as replacements [3]. Bromophenols can also emerge from the degradation of other BFRs and chlorination during water treatment [4–6]. Furthermore, 2,4-DBP, 2,6-dibromophenol (2,6-DBP), and 2,4,6-TBP are thought to be secondary metabolites in marine algae and have been detected in various marine organisms [7–9].

As a result of all these uses, man-made and natural produced bromophenols have been detected to be ubiquitous in environmental media and human biological samples [2]. This, of course, poses serious threats to the environment and human health. 2,4,6-TBP and thyroxine have similar structures; thus, 2,4,6-TBP can regulate thyroid-stimulating hormone (TSH) and disturb the thyroid hormone system in mice [10]. 2,4-DBP, 2,6-DBP, and 2,4,6-TBP have also been reported to disturb cellular Ca^2+^ signaling in neuroendocrine cells (PC12) [11]. Notably, 2,4,6-TBP was found to have stronger toxicity to *Oreochromis niloticus* than decabromodiphenyl ether (BDE-209) through oral exposure [12]. Chlorophenols have been recognized as 2B carcinogenic compounds by the International Agency for Research on Cancer (IARC). By comparison, bromophenols may pose even more serious toxic effects. This is because the leaving tendency of halogens determines the toxicity of halophenols, and bromine (Br) is more potent than chlorine (Cl) [13, 14]. In addition, the Standards for Drinking Water Quality (GB 5749-2022), Environmental Quality Standards for Surface Water (GB 3838-2002), and Quality Standard for Ground Water (GB/T 14848-2017) in China have established chlorophenol environmental control standards. However, few studies exist that have investigated the environmental criteria for bromophenols.

The basis for deriving environmental chemical criteria is to conduct toxicity tests. The general guideline of the Organization for Economic Co-operation and Development (OECD) stipulates that in acute immobilization test of *D. magna*, neonates less than 24 h old are exposed to a series of concentrations of the tested chemical in the culture medium for 48 h [15]. In addition, there is no diet supplied throughout the entire experiment. However, an increasing number of studies have indicated that diet supplement in the test system may help to simulate the real environment [16–20]. Moreover, *D. magna* would grow slowly and accumulate chemicals more easily during starvation [18]. This disconnection between the conditions of laboratory toxicity tests and those of the natural environment may increase the uncertainties in the criteria-setting process for chemicals.

The species sensitivity distribution (SSD) model is proposed to estimate the hazard concentration to protect aquatic organisms from chemicals by using the statistical extrapolation method to fit the distribution of toxicity data of several aquatic species [21]. The concentration that can protect 95% of species is calculated, i.e., the hazardous concentration for 5% species (HC_5_) [22]. However, the current problem is the lack of bromophenol toxicity data. The technical guidance document (TGD) of the European Union (EU) and technical guideline for deriving water quality criteria for freshwater organisms (HJ 831–2002) of China recommend using toxicity data from 10 different species, whereas the United States Environmental Protection Agency (US EPA) requires data from at least eight species [23]. Toxicity prediction models provide a new approach for SSD. The web-based interspecies correlation estimation model (Web-ICE) developed by US EPA can estimate the toxicity of a chemical to various species based on its known toxicity to a surrogate species [24]. Thus, the Web-ICE coupled with SSD model can significantly increase the reliability in hazard concentration prediction.

In this study, the acute toxicities of 2,4-DBP, 2,6-DBP, and 2,4,6-TBP to *Scenedesmus quadricauda* and *Daphnia magna* are revealed based on the standard OECD tests. Additionally, a modified acute toxicity test with the addition of *S*. *quadricauda* as dietary supplementation to *D*. *magna* is proposed to evaluate the toxicity effects of 2,4-DBP, 2,6-DBP, and 2,4,6-TBP. The Web-ICE-SSD model is then applied to estimate the HC_5_ values of 2,4-DBP, 2,6-DBP, and 2,4,6-TBP. The results of this study can provide practical data to support the risk assessment of 2,4-DBP, 2,6-DBP, and 2,4,6-TBP under realistic environmental conditions.

## 2. Materials and Methods

### 2.1. Strains and Culture Conditions

*Scenedesmus quadricauda* is a freshwater green alga, and it was purchased from Freshwater Algae Culture Collection at the Institute of Hydrobiology, Chinese Academy of Sciences (Wuhan, China). It was cultured in the BG-11 medium at 25 ± 0.5°C under 4000 Lux light intensity and 12:12 h photoperiod [25, 26]. The pH value of the culture medium was maintained at 7.0 using 0.1 mmol/L 3-(N-morpholino) propanesulfonic acid (MOPS) [27].

*Daphnia magna* is a water flea that was obtained from Zhejiang Tuokesi Biotechnology Co., Ltd (Zhejiang, China). This aquatic organism was maintained in artificial freshwater (AFW) at 20 ± 0.5°C, under 1500 Lux illumination and 16 h light:8 h dark photoperiod [16]. *D. magna* was fed with *S. quadricauda* once a day, and the entire culture medium was renewed every three days. The culture medium pH was maintained at 7.0 using 0.1 mmol/L MOPS [27].

### 2.2. Acute Toxicity Tests

The growth inhibition test of *S. quadricauda* was conducted according to the OECD testing guideline No. 201 [28]. The logarithmic growth phase of *S. quadricauda* was exposed to a series of 2,4-DBP, 2,6-DBP, and 2,4,6-TBP concentrations (Table 1) in triplicate for 96 h. A total of 50% exposure solution was replaced every 24 h. The cell density was approximately 1.0 × 10^5^ cells/mL at the beginning of the culture system, and it was measured every 24 h using the hemocytometer. The control group was conducted without the additions of 2,4-DBP, 2,6-DBP, or 2,4,6-TBP.

The immobilization test of *D. magna* was begun using neonates (less than 24 h old) with reference to the test guideline of the OECD No. 202 [15]. Briefly, seven first instar daphnids were placed in 50 mL AFW medium that consisted of different 2,4-DBP, 2,6-DBP, and 2,4,6-TBP concentrations (Table 1). A total of 50% exposure solution was replaced every 24 h. The immobilization rate was calculated after 48 h of exposure. Five replicates of each treatment were conducted.

The effects of dietary supplementation on the toxicities of 2,4-DBP, 2,6-DBP, and 2,4,6-TBP to *D. magna* were investigated using a modified test, which conducted the same procedures as described in the OECD testing guideline No. 202 with the additional inclusion of 1.0 × 10^4^ cells/mL inactivated *S. quadricauda* as dietary supplementation. This supplementation could meet the ingestion needs of *D. magna* [20]. During the modified tests, 50% of the exposure solution was replaced every 24 h, and the total exposure duration was set at 48 h according to the standard *D. magna* test.

A 5 mL exposure solution was sampled at the beginning and end of these tests. The solution samples were filtered through 0.45 μm glass fiber filters and stored at −20°C prior to the analysis of 2,4-DBP, 2,6-DBP, and 2,4,6-TBP.

The solid phase extraction coupled with high performance liquid chromatographic method (SPE-HPLC) according to Chi et al. [29] with some modifications was used to determine the 2,4-DBP, 2,6-DBP, and 2,4,6-TBP concentrations in the exposure solution. Detailed experimental procedures are provided in Text S1.

### 2.3. Web-ICE Model Prediction and SSD Curve Generation

The Web-ICE platform (v 3.3) (https://www3.epa.gov/webice/index.html) was used to estimate the acute toxicities of 2,4-DBP, 2,6-DBP, and 2,4,6-TBP on different species, and *S. quadricauda* and *D. magna* were regarded as the surrogate species. The following principles were upheld to reduce the uncertainty of the predicted data: *R*^2^ > 0.6, *p* value < 0.01, mean square error (MSE) < 0.95, cross-validation success > 60%, and slope > 0.6 [30].

The acute toxicity data of *S. quadricauda* and *D. magna* and Web-ICE predicted species were then applied to construct the SSD curves according to our laboratories' previous work [31, 32]. The HC_5_ values of 2,4-DBP, 2,6-DBP, and 2,4,6-TBP as potential thresholds for aquatic organisms were calculated from SSD curves. Methods for generating SSD curves and calculation of HC_5_ values are outlined in Text S2.

### 2.4. Statistical Analysis

The statistical analysis was conducted using SPSS 23.0 software. The median effect concentration (EC_50_) values and the 95% confidence intervals were calculated using the probit analysis. The homogeneity between tested toxicity data and predicted toxicity data was examined using the Kolmogorov–Smirnov (KS) test. The customized program of R v3.6.3 was used to construct SSD curves of 2,4-DBP, 2,6-DBP, and 2,4,6-TBP. The graphical works were generated using Origin 2024.

## 3. Results and Discussion

### 3.1. Acute Toxicities of 2,4-DBP, 2,6-DBP, and 2,4,6-TBP to *S. quadricauda*

**Figure 1:**
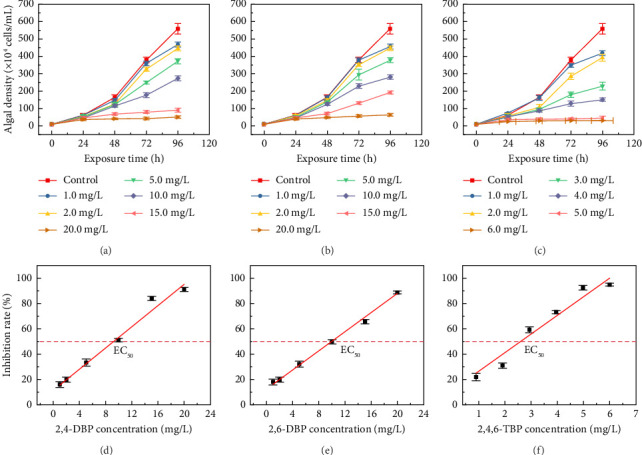
The growth and concentration-response curves of *S. quadricauda* exposed to 2,4-DBP (a, d), 2,6-DBP (b, e), and 2,4,6-TBP (c, f).

The growth performances of *S. quadricauda* exposed to 2,4-DBP, 2,6-DBP, and 2,4,6-TBP are shown in Figures 1(a), 1(b), and 1(c). The cell densities of *S. quadricauda* increased with the increase of exposure time. However, *S. quadricauda* growth was obviously inhibited with the increase in the exposure concentrations of 2,4-DBP, 2,6-DBP, and 2,4,6-TBP. A similar result was observed by the previous study performed in our laboratory. With the 2,4-DBP exposure concentration range of 0.1 to 10.0 mg/L and 2,4,6-TBP range of 0.05 to 5.0 mg/L, the cell densities of *Prorocentrum donghaiense* decreased as the 2,4-DBP and 2,4,6-TBP concentrations rose [33]. Liu et al. [34] also observed that the viability of human extended pluripotent stem cells decreased with increasing 2,4,6-TBP exposure concentrations.

The 2,4-DBP, 2,6-DBP, and 2,4,6-TBP tested concentrations in the exposure solution of *S. quadricauda* at the beginning and end of the acute toxicity tests are summarized in Table S1. The deviations were within 20% in all exposure solutions between 0 and 96 h. Hence, the nominal concentrations were used in fitting EC_50_ values of *S. quadricauda*. The concentration-response curves and the EC_50_ values of these three bromophenols are shown in Figures 1(d), 1(e), and 1(f) and Table 2. The acute toxicity order was 2,4,6-TBP (2.67 mg/L) > 2,4-DBP (8.73 mg/L) > 2,6-DBP (9.90 mg/L) according to the calculated EC_50_ values. Sazawa et al. reported that the EC_50_ value of 2,6-DBP for *Pseudokirchneriella subcapitata* was 49 mg/L, which indicated that 2,6-DBP had different toxic effects on different species [44]. For organisms of the same genus as in this study, the EC_50_ values of 2,4-dichlorophenol (2,4-DCP) and 2,4,6-trichlorophenol (2,4,6-TCP) for *Scenedesmus obliquus* were 9.76 and 2.46 mg/L, respectively [27]. This result indicated that the toxicities of chlorophenols to *S. obliquus* were comparable to those of bromophenols to *S. quadricauda*.

### 3.2. Acute Toxicities of 2,4-DBP, 2,6-DBP, and 2,4,6-TBP to *D. magna*

**Figure 2:**
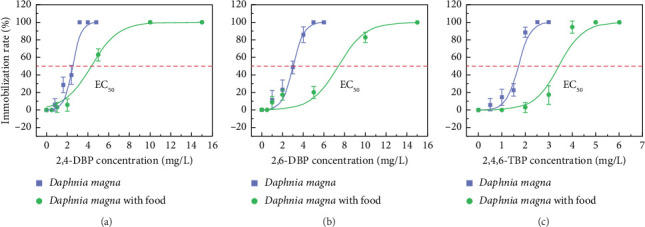
The concentration-response curves of 2,4-DBP (a), 2,6-DBP (b), and 2,4,6-TBP (c) to *D. magna*.

The concentration-response curves of *D. magna* are shown in Figure 2. The tested concentrations of *D. magna* exposure solution are listed in Tables S2 and S3. In agreement with what is stated in Section 3.1, the nominal concentrations listed in Table 1 were also used in the related calculations of the EC_50_ values of *D. magna*. The results suggested that 2,4,6-TBP (EC_50_ = 1.57 mg/L) was the most toxic chemical among the three bromophenols (EC_50, 2,4-DBP_ = 2.17 mg/L, EC_50, 2,6-DBP_ = 2.78 mg/L). This finding was comparable to that of Kopperman et al. [45] who reported that the EC_50_ value of 2,4,6-TBP to *D. magna* was 1.31 mg/L. Furthermore, *D. magna* was more sensitive to 2,4-DBP, 2,6-DBP, and 2,4,6-TBP compared with *S. quadricauda*. Among *Tetradesmus obliquus*, *D. magna*, and *D. rerio*, *D. magna* was the most sensitive species to 2,2-bis(4-hydroxyphenyl)butane (BPB) [46]. Yang et al. [47] also observed that *D. magna* was the most sensitive species to polychlorinated diphenyl ether (PCDE) exposure compared with *S. obliquus* and *D*. *rerio*.

As shown in Figure 2, the addition of *S. quadricauda* as dietary supplementation significantly alleviated the toxicity effects of bromophenols to *D. magna*. Specifically, the EC_50_ values showed marked elevations: 2,4-DBP increased from 2.17 to 4.47 mg/L, 2,6-DBP from 2.78 to 6.75 mg/L, and 2,4,6-TBP increased from 1.57 to 3.28 mg/L. An earlier study had also suggested that the toxicities of Cu and Zn to *D. magna* were obviously reduced by dietary supplementation [16]. Algae can provide metabolic energy for *Daphnia* and effectively ease toxicity stress. Tawfeek et al. [48] also confirmed that *Chlorella vulgaris* supplementation alleviated the oxidative stress in Nile tilapia (*O. niloticus*) induced by chlorpyrifos (CPF). Hylton et al. [17] and Stevenson et al. [18] suggested that dietary supplements during acute toxicity tests may conform with environmental realism. However, the correct amount of supplement addition remains inconclusive and requires further study. The culture system may become more complex with the increase of algal density due to the interaction between algae and compounds, as evidenced in bioaccumulation and biodegradation processes.

### 3.3. Toxicity Comparisons of 2,4-DBP, 2,6-DBP, and 2,4,6-TBP

**Figure 3:**
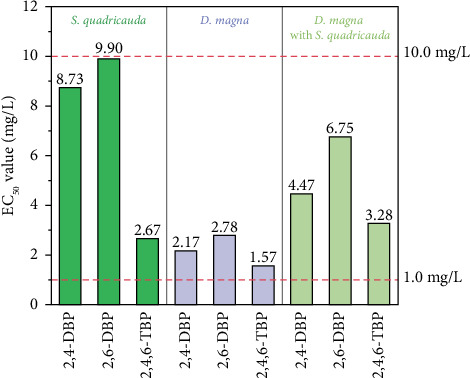
The EC_50_ values of 2,4-DBP, 2,6-DBP, and 2,4,6-TBP in the study.

Figure 3 shows the toxic ranking of the three bromophenols which was found to be 2,4,6-TBP > 2,4-DBP > 2,6-DBP in the tests. The acute toxicities of these three bromophenols increased with the increase in substituted bromine atoms. Schultz et al. [49] observed that the EC_50_ values of 2,4,6-TBP and 2,4-DBP to *Tetrahymena pyriformis* were 2.95 and 9.97 mg/L, respectively. Similar results were also investigated in chlorophenols, and 2,4-DCP was more toxic than 3-chlorophenol (3-CP) [50]. However, Yang et al. [51] concluded the opposite; they found that the rank order of bromophenols toxicity to *Platynereis dumerilii* was 4-bromophenol (4-BP) > 2,4-DBP > 2,4,6-TBP. This may be attributed to the different pH values of the exposure solution; the pH value of the culture medium in their study was 8.0. However, in this study, it was 7.0. Phenolic hydroxyl group can be protonated or ionized based on different pH values [27, 51]. Such changes in its chemical state directly influence the bioaccumulation of bromophenols in test organisms.

According to the Guidelines for the Hazard Evaluation of New Chemical Substances (HJ/T154-2004) (Table S4), 2,4-DBP, 2,6-DBP, and 2,4,6-TBP are in the “Toxic” category (1.0 < EC_50_ < 10.0 mg/L). The acute toxicity data of 2,4-DCP, 2,6-dichlorophenol (2,6-DCP), and 2,4,6-TCP are summarized in Table 2. It can be seen that the acute toxicities between chlorophenols and bromophenols are comparable. This similarity in acute toxicity thresholds may arise from their shared phenolic structure and analogous modes of action under high-concentration, short-term exposure scenarios. However, the toxicokinetic differences may become more significant under low-concentration, long-term exposure scenarios. Specifically, bromine exhibits a larger atomic radius and lower electronegativity than chlorine, which can enhance the lipophilicity and bioaccumulation potential of bromophenols [52]. Xiao et al. [53] reported that the permeability values of 2,4-DBP and 2,4-DCP across human skin were 0.031 and 0.021 cm/h, respectively, suggesting a higher dermal absorption risk for bromophenols.

In addition, the water quality criteria of chlorophenols have been studied by various researchers [54–59]. 2,4-DCP, 2,6-DCP, and 2,4,6-TCP have been regarded as priority pollutants in China, the United States, the IARC, and the World Health Organization (WHO). In contrast, despite their extensive application in BFRs, disinfectants, and antiseptic agents, bromophenols have received relatively little research attention [2]. Furthermore, emerging evidence has displayed that bromophenols can form dioxin-like compounds under biotic and abiotic conditions [33, 60]. This discrepancy suggests an urgent need to establish environmental quality standards and monitoring programs for bromophenols.

### 3.4. Aquatic Life Criteria of 2,4-DBP, 2,6-DBP, and 2,4,6-TBP

The SSD method assumes that the toxicity data of selected species can represent the toxic threshold of the entire ecosystem. However, the toxicity data of bromophenol are scarce; hence, the fitting uncertainty of SSD model will increase. The Web-ICE model can be used to provide additional data to ensure the accuracy of SSD model [61]. Following the screening principles proposed by Shen et al. [30], 21 predicted species were selected using *S. quadricauda* and *D. magna* as the surrogate species. The statistical parameters between surrogate species and predicted species are shown in Table S5. The selected ICE models exhibited strong reliability in toxicity extrapolation, with correlation coefficients (*R*^2^) > 0.6, *p* values < 0.01, MSE < 0.95, and cross-validation success rates > 60% [62, 63]. The predicted toxicity data of 2,4-DBP, 2,6-DBP, and 2,4,6-TBP are listed in Tables S6 and S7. Additionally, the KS test was used to test data consistency between the experimental data and the predicted data. As shown in Table S8, there was no significant difference between the experimental data and the predicted data (*p* > 0.05), which means that the toxicity data derived from the Web-ICE model were valid.

**Figure 4:**
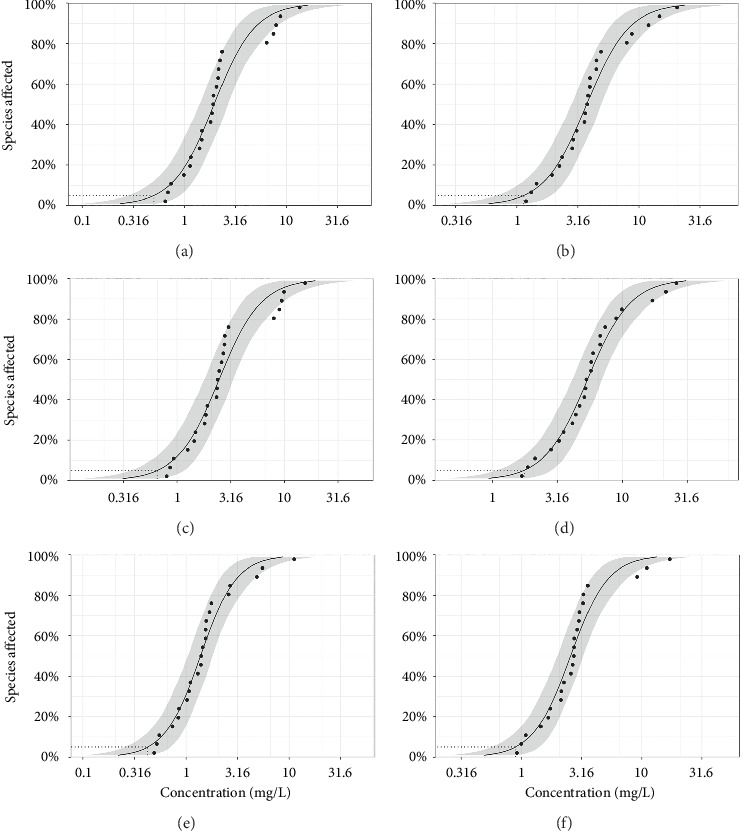
SSD curves of 2,4-DBP (a, b), 2,6-DBP (c, d), and 2,4,6-TBP (e, f). (a), (c), and (e) were generated from standard toxicity data and their predicted data; (b), (d), and (f) were generated from modified toxicity data and their predicted data.

Figure 4 shows that two sets of SSD curves were generated based on the standard toxicity tests (Figures 4(a), 4(c), and 4(e)) and the modified toxicity tests (Figures 4(b), 4(d), and 4(f)). As listed in Table 3, HC_5_ values of 2,4-DBP, 2,6-DBP, and 2,4,6-TBP were 0.55, 0.71, and 0.43 mg/L derived from the standard tests, respectively. Correspondingly, the HC_5_ values of these three bromophenols calculated from the modified tests were 1.20, 1.80, and 0.88 mg/L, respectively. Notably, the HC_5_ values of 2,4-DCP and 2,4,6-TCP reported by Xing et al. [27] were 0.95 and 0.74 mg/L, respectively, which were slightly higher than those of 2,4-DBP and 2,4,6-TBP derived from standard tests in the study. The above results suggested that these bromophenols may pose comparable environmental risks to chlorophenols, suggesting the need for similar regulatory control. Additionally, the HC_5_ values calculated from standard tests were smaller compared to those of the modified tests, which indicated that the standard tests may overestimate the environmental risk because the modified method more closely fit the actual eco-environment [64]. Such overestimation highlights the importance of adopting ecologically relevant tests for chemical risk assessments. Furthermore, the higher HC_5_ values from the modified tests can reduce industrial compliance costs without compromising ecosystem protection. Integrating modified tests into regulatory guidelines would enhance the accuracy of risk evaluations while avoiding unnecessary economic burdens.

## 4. Conclusion

According to the EC_50_ values, 2,4-DBP, 2,6-DBP, and 2,4,6-TBP were toxic to *S. quadricauda and D. magna.* In addition, *D. magna* was more sensitive to bromophenols compared with *S. quadricauda.* The results also showed that dietary supplementation reduced the toxicity effects of bromophenols to aquatic life. It may be important to consider dietary supplementation when conducting toxicity tests, as this would be more representative of the actual environment. Future studies should investigate the long-term exposure of bromophenols to determine their potential impacts on growth, reproduction, and offspring viability. Additionally, further investigation is needed to clarify the role of dietary supplementation in mitigating cumulative toxicity, focusing on oxidative damage or metabolic pathways. Such findings could provide critical insights for ecological risk assessments of bromophenols in natural ecosystems.

## Figures and Tables

**Table 1:** The nominal exposure concentrations used in the acute toxicity tests.

Chemicals	Tests	Concentrations (mg/L)					
		1	2	3	4	5	6
2,4-DBP	*S. quadricauda*	1.0	2.0	5.0	10.0	15.0	20.0
	*D. magna*	0.8	1.6	2.4	3.2	4.0	4.8
	*D. magna* with dietary	0.5	1.0	2.0	5.0	10.0	15.0

2,6-DBP	*S. quadricauda*	1.0	2.0	5.0	10.0	15.0	20.0
	*D. magna*	1.0	2.0	3.0	4.0	5.0	6.0
	*D. magna* with dietary	0.5	1.0	2.0	5.0	10.0	15.0

2,4,6-TBP	*S. quadricauda*	1.0	2.0	3.0	4.0	5.0	6.0
	*D. magna*	0.5	1.0	1.5	2.0	2.5	3.0
	*D. magna* with dietary	1.0	2.0	3.0	4.0	5.0	6.0

*Note:* The exposure concentrations were set according to the pretests.

**Table 2:** EC_50_ values of 2,4-DCP, 2,6-DCP, and 2,4,6-TCP.

Chemicals	Aquatic species	Group	Observed duration (days)	EC_50_ values (mg/L)	References
2,4-DCP	*Raphidocelis subcapitata*	Algae	4	14.00	[35]
	*Chlorella vulgaris*	Algae	4	9.20	[35]
	*Skeletonema costatum*	Algae	4	8.03	[36]
	*Gammarus pulex*	Crustaceans	2	2.48	[37]
	*Daphnia magna*	Crustaceans	2	3.68	[38]

2,6-DCP	*Chlorella vulgaris*	Algae	4	9.70	[35]
	*Raphidocelis subcapitata*	Algae	4	29.00	[35]
	*Daphnia magna*	Crustaceans	2	3.40	[39]

2,4,6-TCP	*Raphidocelis subcapitata*	Algae	4	3.50	[35]
	*Chlorella vulgaris*	Algae	4	3.20	[40]
	*Scenedesmus abundans*	Algae	4	5.60	[41]
	*Ceriodaphnia dubia*	Crustaceans	2	4.00	[42]
	*Daphnia magna*	Crustaceans	2	1.71	[43]

**Table 3:** HC_5_ values of 2,4-DBP, 2,6-DBP, and 2,4,6-TBP derived from SSD curves.

Chemicals	HC_5_ (95% confidence intervals) (mg/L)	
	Standard tests	Modified tests
2,4-DBP	0.55 (0.34–0.96)	1.20 (0.79–1.94)
2,6-DBP	0.71 (0.45–1.22)	1.80 (1.20–2.85)
2,4,6-TBP	0.43 (0.28–0.72)	0.88 (0.59–1.39)

## Data Availability

The data that support the findings of this study are available from the corresponding author upon reasonable request.

## Supporting Information

Text S1: Exposure solution pretreatment and instrumental analysis. Text S2: Construction of SSD curves and calculation of HC_5_ values. Table S1: Monitoring results of 2,4-DBP, 2,6-DBP, and 2,4,6-TBP in the acute test of *Scenedesmus quadricauda*. Table S2: Monitoring results of 2,4-DBP, 2,6-DBP, and 2,4,6-TBP in the acute test of *Daphnia magna*. Table S3: Monitoring results of 2,4-DBP, 2,6-DBP, and 2,4,6-TBP in the acute test of *Daphnia magna* with food. Table S4: The evaluation criteria of chemicals. Table S5: Statistical parameters of ICE model. Table S6: Predicted toxicity data of standard tests using ICE models. Table S7: Predicted toxicity data of modified tests using ICE models. Table 8: The results of KS test for toxicity data of 2,4-DBP, 2,6-DBP, and 2,4,6-TBP.
