# Supplementary material for: Acute Toxicities of Bromophenols to Alga and Daphina: Comparative Species Sensitivity Distribution Between Standard and Dietary Supplementation Tests
Source: J Toxicol. 2025 May 11;2025:3399746. doi: 10.1155/jt/3399746 (PMC12086033; doi:10.1155/jt/3399746)
Supplement: Supporting Information — Additional supporting information can be found online in the Supporting Information section. [file 3399746.f1.docx]

Text S1. Exposure solution pretreatment and instrumental analysis

The Poly-Sery HLB Pro cartridges (200 mg, 6 mL) were preconditioned with 7 mL methanol (MeOH) and 7 mL ultrapure water (UPW). The sampled exposure solution was then loaded on the cartridges. A total of 10 mL UPW and 5 mL MeOH: UPW (6: 4, *v*: *v*) were used to wash the columns in sequence. The analytes were eluted with 6 mL MeOH and evaporated to nearly dryness under the nitrogen flow at 50 °C after 10 min vacuum drying treatment. Finally, the concentrated extracts were reconstituted in 1 mL MeOH for HPLC analysis using the autosampler and the UV detector (U3000, Thermo Fisher Scientific Inc., Germany).

Twenty microliters of each prepared extract were injected into a Thena C18 column (150 mm × 4.6 mm × 5 μm) at a flow rate of 1.0 mL/ min at 30 °C. The mobile phase consisted of 80% MeOH and 20% UPW. Additionally, 2,4-DBP and 2,6-DBP were measured at 280 nm and 2,4,6-TBP at 290 nm.

Text S2. Construction of SSD curves and calculation of HC_5_ values

The toxicity data for each bromophenol were ranked using an ascending numerical sort, and the percent of species affected was calculated using the following equation:

Percent of species affected = (*i* - 0.5)/*N*

In the formula, *i* is the ordinal number in the collated data and *N* is the total amount of toxicity data. Then, the SSD curves were created with the toxicity values as the X-axis and percent of species affected as the Y-axis, and fitted with log-normal model. The HC_5_ value was calculated as the concentration affecting 5% of species.

Table S1. Monitoring results of 2,4-DBP, 2,6-DBP and 2,4,6-TBP in the acute test of *Scenedesmus quadricauda*

| Chemicals | Nominal concentrations  (mg/L) | Tested concentrations (mg/L) | |
| --- | --- | --- | --- |
|  |  | 0 h | 96 h |
| 2,4-DBP | 1.0 | 1.21 ± 0.42 | 0.85 ± 0.16 |
|  | 10.0 | 9.24 ± 0.35 | 7.00 ± 0.21 |
|  | 20.0 | 18.85 ± 0.48 | 15.45 ± 0.80 |
| 2,6-DBP | 1.0 | 1.39 ± 0.37 | 1.03 ± 0.24 |
|  | 10.0 | 9.91 ± 0.28 | 8.24 ± 0.49 |
|  | 20.0 | 18.70 ± 0.63 | 14.58 ± 1.01 |
| 2,4,6-TBP | 1.0 | 1.33 ± 0.51 | 0.96 ± 0.73 |
|  | 4.0 | 4.11 ± 0.82 | 3.12 ± 0.53 |
|  | 6.0 | 6.20 ± 1.06 | 5.33 ± 0.87 |

Table S2. Monitoring results of 2,4-DBP, 2,6-DBP and 2,4,6-TBP in the acute test of *Daphnia magna*

| Chemicals | Nominal concentrations  (mg/L) | Tested concentrations (mg/L) | |
| --- | --- | --- | --- |
|  |  | 0 h | 48 h |
| 2,4-DBP | 0.8 | 0.89 ± 0.12 | 0.73 ± 0.47 |
|  | 3.2 | 3.23 ± 0.13 | 2.85 ± 0.64 |
|  | 4.8 | 4.73 ± 0.35 | 4.41 ± 0.96 |
| 2,6-DBP | 1.0 | 0.94 ± 0.48 | 0.71 ± 0.33 |
|  | 4.0 | 4.33 ± 0.36 | 3.69 ± 0.24 |
|  | 6.0 | 6.29 ± 0.30 | 5.52 ± 0.43 |
| 2,4,6-TBP | 0.5 | 0.56 ± 0.62 | 0.43 ± 0.64 |
|  | 2.0 | 2.20 ± 0.54 | 2.01 ± 0.59 |
|  | 3.0 | 3.08 ± 0.81 | 2.70 ± 0.44 |

Table S3. Monitoring results of 2,4-DBP, 2,6-DBP and 2,4,6-TBP in the acute test of *Daphnia magna* with food

| Chemicals | Nominal concentrations  (mg/L) | Tested concentrations (mg/L) | |
| --- | --- | --- | --- |
|  |  | 0 h | 48 h |
| 2,4-DBP | 0.5 | 0.52 ± 0.64 | 0.35 ± 0.55 |
|  | 5.0 | 5.79 ± 0.42 | 4.34 ± 0.30 |
|  | 15.0 | 15.59 ± 0.23 | 11.54 ± 0.21 |
| 2,6-DBP | 0.5 | 0.48 ± 0.31 | 0.29 ± 0.52 |
|  | 5.0 | 5.31 ± 0.26 | 4.38 ± 0.23 |
|  | 15.0 | 15.24 ± 0.28 | 13.26 ± 0.45 |
| 2,4,6-TBP | 1.0 | 0.99 ± 0.65 | 0.70 ± 0.53 |
|  | 4.0 | 3.84 ± 0.31 | 2.86 ± 0.49 |
|  | 6.0 | 6.05 ± 0.25 | 4.60 ± 0.24 |

Table S4. The evaluation criteria of chemicals

| Toxicity range (mg/L) | Classification |
| --- | --- |
| EC_50_/ LC_50_< 1 | Very toxic |
| 1< EC_50_/ LC_50_< 10 | Toxic |
| 10< EC_50_/ LC_50_< 100 | Harmful |
| EC_50_/ LC_50_> 100 | Not harmful |

Table S5. Statistical parameters of ICE model

|  | Predicted species | Surrogate species | R^2^ | *p* value | Mean square error  (MSE) | Cross-validation success  (%) | Slope |
| --- | --- | --- | --- | --- | --- | --- | --- |
| 1 | *Desmodesmus subspicatus* | *Scenedesmus quadricauda* | 0.78 | 0.00 | 0.50 | 78 | 0.94 |
| 2 | *Thamnocephalus platyurus* | *Daphnia magna* | 0.99 | 0.00 | 0.06 | 91 | 0.92 |
| 3 | *Ceriodaphnia dubia* | *Daphnia magna* | 0.96 | 0.00 | 0.27 | 81 | 1.00 |
| 4 | *Daphnia pulex* | *Daphnia magna* | 0.97 | 0.00 | 0.12 | 90 | 1.01 |
| 5 | *Simocephalus serrulatus* | *Daphnia magna* | 0.88 | 0.00 | 0.21 | 87 | 1.00 |
| 6 | *Lampsilis siliquoidea* | *Daphnia magna* | 0.86 | 0.00 | 0.47 | 71 | 0.75 |
| 7 | *Paratanytarsus parthenogeneticus* | *Daphnia magna* | 0.99 | 0.00 | 0.04 | 100 | 0.94 |
| 8 | *Oreochromis mossambicus* | *Daphnia magna* | 0.76 | 0.00 | 0.34 | 82 | 0.57 |
| 9 | *Americamysis bahia* | *Daphnia magna* | 0.68 | 0.00 | 0.94 | 64 | 0.84 |
| 10 | *Metamysidopsis insularis* | *Daphnia magna* | 0.94 | 0.01 | 0.18 | 80 | 0.86 |
| 11 | *Lampsilis rafinesqueana* | *Daphnia magna* | 0.98 | 0.01 | 0.07 | 100 | 0.98 |
| 12 | *Utterbackia imbecillis* | *Daphnia magna* | 0.97 | 0.00 | 0.12 | 100 | 0.90 |
| 13 | *Actinonaias pectorosa* | *Daphnia magna* | 0.97 | 0.01 | 0.14 | 75 | 1.00 |
| 14 | *Lymnaea stagnalis* | *Daphnia magna* | 0.96 | 0.00 | 0.19 | 78 | 1.01 |
| 15 | *Physa gyrina* | *Daphnia magna* | 0.97 | 0.00 | 0.15 | 89 | 0.99 |
| 16 | *Amblema plicata* | *Daphnia magna* | 0.95 | 0.00 | 0.19 | 90 | 0.88 |
| 17 | *Branchinecta lynchi* | *Daphnia magna* | 0.98 | 0.00 | 0.09 | 100 | 0.90 |
| 18 | *Megalonaias nervosa* | *Daphnia magna* | 0.96 | 0.00 | 0.16 | 91 | 0.93 |
| 19 | *Pseudosida ramosa* | *Daphnia magna* | 0.87 | 0.01 | 0.58 | 67 | 0.93 |
| 20 | *Margaritifera falcata* | *Daphnia magna* | 0.96 | 0.00 | 0.15 | 90 | 0.86 |
| 21 | *Lasmigona complanata* | *Daphnia magna* | 0.98 | 0.00 | 0.11 | 100 | 0.92 |

Note: For best estimates, models were selected that possessed the following: 1. R^2^> 0.6; 2. *p*< 0.01; 3. MSE< 0.95; 4. Cross-validation success> 60%; 5. Slope> 0.6.

Table S6. Predicted toxicity data of standard tests using ICE models

|  | Prediction | Surrogate | Predicted toxicity values (mg/L) | | |
| --- | --- | --- | --- | --- | --- |
|  |  |  | 2,4-DBP | 2,6-DBP | 2,4,6-TBP |
| 1 | *Desmodesmus subspicatus* | *S. quadricauda* | 7.93 | 8.94 | 2.58 |
| 2 | *Thamnocephalus platyurus* | *D. magna* | 1.87 | 2.35 | 1.39 |
| 3 | *Ceriodaphnia dubia* | *D. magna* | 1.49 | 1.91 | 1.07 |
| 4 | *Daphnia pulex* | *D. magna* | 1.81 | 2.33 | 1.30 |
| 5 | *Simocephalus serrulatus* | *D. magna* | 2.15 | 2.76 | 1.55 |
| 6 | *Lampsilis siliquoidea* | *D. magna* | 2.24 | 2.69 | 1.76 |
| 7 | *Paratanytarsus parthenogeneticus* | *D. magna* | 7.47 | 9.42 | 5.51 |
| 8 | *Oreochromis mossambicus* | *D. magna* | 13.48 | 15.56 | 11.18 |
| 9 | *Americamysis bahia* | *D. magna* | 0.65 | 0.80 | 0.49 |
| 10 | *Metamysidopsis insularis* | *D. magna* | 6.41 | 7.94 | 4.86 |
| 11 | *Lampsilis rafinesqueana* | *D. magna* | 1.16 | 1.48 | 0.84 |
| 12 | *Utterbackia imbecillis* | *D. magna* | 1.48 | 1.85 | 1.10 |
| 13 | *Actinonaias pectorosa* | *D. magna* | 1.41 | 1.80 | 1.02 |
| 14 | *Lymnaea stagnalis* | *D. magna* | 2.34 | 3.01 | 1.69 |
| 15 | *Physa gyrina* | *D. magna* | 1.93 | 2.47 | 1.40 |
| 16 | *Amblema plicata* | *D. magna* | 0.69 | 0.86 | 0.52 |
| 17 | *Branchinecta lynchi* | *D. magna* | 2.07 | 2.59 | 1.55 |
| 18 | *Megalonaias nervosa* | *D. magna* | 1.14 | 1.44 | 0.85 |
| 19 | *Pseudosida ramosa* | *D. magna* | 0.74 | 0.93 | 0.55 |
| 20 | *Margaritifera falcata* | *D. magna* | 1.92 | 2.38 | 1.45 |
| 21 | *Lasmigona complanata* | *D. magna* | 0.99 | 1.25 | 0.74 |

Table S7. Predicted toxicity data of modified tests using ICE models

|  | Predicted taxa | Surrogate | Predicted toxicity values (mg/L) | | |
| --- | --- | --- | --- | --- | --- |
|  |  |  | 2,4-DBP | 2,6-DBP | 2,4,6-TBP |
| 1 | *Desmodesmus subspicatus* | *S. quadricauda* | 7.93 | 8.94 | 2.58 |
| 2 | *Thamnocephalus platyurus* | *D. magna* with food | 3.63 | 5.29 | 2.73 |
| 3 | *Ceriodaphnia dubia* | *D. magna* with food | 3.09 | 4.68 | 2.26 |
| 4 | *Daphnia pulex* | *D. magna* with food | 3.77 | 5.74 | 2.75 |
| 5 | *Simocephalus serrulatus* | *D. magna* with food | 4.46 | 6.76 | 3.26 |
| 6 | *Lampsilis siliquoidea* | *D. magna* with food | 3.83 | 5.21 | 3.04 |
| 7 | *Paratanytarsus parthenogeneticus* | *D. magna* with food | 14.71 | 21.65 | 11.00 |
| 8 | *Oreochromis mossambicus* | *D. magna* with food | 20.49 | 26.02 | 17.13 |
| 9 | *Americamysis bahia* | *D. magna* with food | 1.19 | 1.68 | 0.92 |
| 10 | *Metamysidopsis insularis* | *D. magna* with food | 11.95 | 17.03 | 9.15 |
| 11 | *Lampsilis rafinesqueana* | *D. magna* with food | 2.35 | 3.52 | 1.74 |
| 12 | *Utterbackia imbecillis* | *D. magna* with food | 2.84 | 4.12 | 2.15 |
| 13 | *Actinonaias pectorosa* | *D. magna* with food | 2.90 | 4.38 | 2.13 |
| 14 | *Lymnaea stagnalis* | *D. magna* with food | 4.87 | 7.39 | 3.56 |
| 15 | *Physa gyrina* | *D. magna* with food | 3.95 | 5.95 | 2.91 |
| 16 | *Amblema plicata* | *D. magna* with food | 1.31 | 1.87 | 1.00 |
| 17 | *Branchinecta lynchi* | *D. magna* with food | 3.97 | 5.76 | 3.01 |
| 18 | *Megalonaias nervosa* | *D. magna* with food | 2.23 | 3.26 | 1.67 |
| 19 | *Pseudosida ramosa* | *D. magna* with food | 1.45 | 2.13 | 1.09 |
| 20 | *Margaritifera falcata* | *D. magna* with food | 3.58 | 5.10 | 2.74 |
| 21 | *Lasmigona complanata* | *D. magna* with food | 1.94 | 2.83 | 1.45 |

Table S8. The results of K-S test for toxicity data of 2,4-DBP, 2,6-DBP and 2,4,6-TBP

| Classification | Chemicals | n_1_^*^ | n_2_^*^ | K-S | *p* |
| --- | --- | --- | --- | --- | --- |
| Experimental toxicity data of standard tests and predicted toxicity data from ICE models | 2,4-DBP | 2 | 21 | 0.97 | 0.31 |
|  | 2,6-DBP | 2 | 21 | 1.03 | 0.24 |
|  | 2,4,6-TBP | 2 | 21 | 0.97 | 0.31 |
| Experimental toxicity data of modified tests and predicted toxicity data from ICE models | 2,4-DBP | 2 | 21 | 1.03 | 0.24 |
|  | 2,6-DBP | 2 | 21 | 0.97 | 0.31 |
|  | 2,4,6-TBP | 2 | 21 | 0.64 | 0.80 |

Notes: n_1_: numbers of experimental toxicity data; n_2_: numbers of predicted toxicity data
